# Impact of transformation on the survival of patients diagnosed with follicular lymphoma that progressed within 24 months

**DOI:** 10.7150/jca.54434

**Published:** 2021-03-05

**Authors:** Sang Eun Yoon, Junhun Cho, Won Seog Kim, Seok Jin Kim

**Affiliations:** 1 Division of Hematology-Oncology, Department of Medicine, Samsung Medical Center, Sungkyunkwan University School of Medicine, Seoul, Korea.; 2 Department of Pathology, Samsung Medical Center, Sungkyunkwan University School of Medicine, Seoul, Korea.

**Keywords:** follicular lymphoma, early progression, transformation

## Abstract

**Background:** Progression within 24 months after initiating treatment (POD24) is established as an unfavorable event predicting poor prognosis in patients with follicular lymphoma (FL). However, little is known about the impact of transformation on the outcome of FL patients with POD24 although transformation could be related to early progression and poor prognosis in FL patients.

**Methods:** We investigated the occurrence of transformation and its association with POD24 in FL patients receiving RCVP (rituximab, cyclophosphamide, vincristine and predisone, n = 152), RCHOP (rituximab, cyclophosphamide, doxorubicin, vincristine and predisone, n = 111), and BR (bendamustine, rituximab, n = 61).

**Results:** With the median follow-up of 48.3 months, disease progression occurred in 94 patients (94/324, 29.0%) including 58 POD24 cases (58/324, 17.9%), and POD24 was more frequent in the RCVP (25/152, 16.4%) and RCHOP (28/111, 25.2%) groups than the BR group (5/61, 8.2%). Transformation was documented in 38 cases, including 22 of which were clinically designated as transformation. Among the 58 cases with POD24, the proportion with transformation differed across groups: RCVP (8/25, 32%); RCHOP (16/28, 57.1%); and BR (5/5, 100%). Transformation accounted for 50% (29/58) of POD24 cases whereas only 9 (9/36, 25%) patients had transformation with progression after 24 months. Patients with transformation within 24 months had the worst survival outcome regardless of POD24.

**Conclusions:** Transformation negatively impacted survival among FL patients more than POD24 itself. With caution, our findings suggest that BR may reduce POD24 more than RCVP or RCHOP. However, BR efficacy may not reduce the occurrence of transformation.

## Introduction

Follicular lymphoma (FL) is the most common indolent non-Hodgkin lymphoma (NHL), and its treatment outcome has been improved with rituximab-containing immunochemotherapy 1-3. As a result, immunochemotherapy regimens, such as rituximab, cyclophosphamide, doxorubicin, vincristine, and prednisone (RCHOP), and rituximab, cyclophosphamide, vincristine, and prednisone (RCVP) are considered the standard of care for the frontline treatment of patients with symptomatic, advanced-stage FL 4, 5. However, most patients still show a relapsing and remitting pattern of illness that requires various salvage treatments. Thus, achieving prolonged progression- and treatment-free survival may be the ultimate goal for FL management. Bendamustine and rituximab (BR) have also been used as a frontline treatment for patients with advanced-stage FL, with two randomized trials demonstrating that BR is not inferior to RCHOP or RCVP in terms of survival outcomes and toxicity profiles 6, 7. In addition, rituximab maintenance has been widely used following frontline treatment because the progression-free survival (PFS) benefits of rituximab maintenance were demonstrated in patients with FL in a phase 3 trial (i.e., PRIMA study) 8, 9. Nevertheless, early relapse or progression remains a problematic issue because these patients were reported to show poorer prognosis than those with late relapse 10. Indeed, this early so-called “progression within 24 months” (POD24), defined as disease progression within 24 months after treatment, was proposed as a prognostic indicator for FL 10, 11. Thus, relapse within 24 months among patients with FL has emerged as an important treatment issue 12. However, not all patients with POD24 follow an aggressive clinical course that results in poor outcomes because asymptomatic progression is possible during follow-up or rituximab maintenance, and some patients with POD24 show prognosis similar to that of patients with late disease progression.

Histological transformation to diffuse large B-cell lymphoma (DLBCL) or lymphomas resembling Burkitt lymphoma (BL) or lymphomas with features intermediate between DLBCL and BL can occur in FL patients with disease progression 13. Transformation is a rare but critical event in FL patients because most patients with transformed FL become refractory to salvage treatments. Although transformation should be confirmed by biopsy, this is not always possible in clinical practice. Therefore, transformation is often clinically suspected when patients show rapid increasing tumor mass or rising serum lactate dehydrogenase (LDH) levels, as revealed in a population-based analysis of transformed NHL 14. Given that transformation occurrence may be related to early progression, prognosis among patients with FL might be more influenced by transformation than by POD24 itself. However, the precise rate of transformation in POD24 is unknown, especially in Asian patients with FL who receive immunochemotherapy such as BR. Thus, we performed a single-center retrospective study to analyze the outcome of patients with FL receiving BR, RCHOP, or RCVP, with or without rituximab maintenance in a real-world context, to investigate the occurrence of transformation and its impact on survival among FL patients with POD24.

## Methods

### Patients

This was a retrospective study of treatment outcomes among patients with newly diagnosed, treatment-naïve, grade 1-3A FL between March 2003 and February 2019. The study sample was selected from two prospective cohort studies (2008-2011, NCT#00822731 and 2012-2016, NCT#01877109) and the lymphoma registry of Samsung Medical Center between 2003 and 2019. The primary study objective was to compare the frequency of transformation according to the frontline treatment type. Transformation was pathologically defined as the presence of large cells on tumor tissue obtained from the biopsy of relapsed sites compatible with DLBCL, or lymphomas resembling BL or with features intermediate between DLBCL and BL 13. However, because biopsy of involved sites was not always possible in patients with relapse or progression, patients with clinically suspected transformation were also designated as transformation based on a high index of clinical suspicion used in a population-based analysis of transformed NHL 14: (1) sudden elevation of LDH (≥2 × upper normal limit); (2) newly developed hypercalcemia; (3) rapid lymph node enlargement confirmed by imaging or physical examination; (4) unusual extranodal involvement such as liver, bone, muscle, and brain at the time of relapse or progression. Second, we analyzed the association between transformation and POD24, designated as relapse or progression, death from lymphoma, or treatment-related toxicity within 24 months after initiation of frontline treatment with BR, RCVP, or RCHOP. We also investigated the impact of transformation on survival among FL patients with and without POD24, and the risk factors for the occurrence of transformation using clinical and laboratory parameters obtained at diagnosis (i.e., before frontline treatment). The inclusion criteria were: (1) pathological diagnosis with FL grades 1, 2, or 3A; (2) symptomatic stage III/IV or bulky localized mass or lymphoma-related cytopenia requiring systemic treatment; (3) receive at least one cycle of induction treatments among three regimens: RCVP, RCHOP, or BR; and (4) have at least 24 months of follow-up data for the analysis of POD24. The exclusion criteria were: (1) FL grade 3B; (2) FL combined with DLBCL at diagnosis; (3) initial treatment with regimens other than BR, RCVP, or RCHOP; (4) being observed without any treatment; (5) pediatric patients.

### Case report forms and treatments

The case report form contained a fill-in section on patient characteristics such as sex, age, Eastern Cooperative Oncology Group performance status, Ann Arbor stage, bone marrow involvement, and crucial laboratory data, including complete blood cell counts and serum LDH. The prognostic risk was calculated using the Follicular Lymphoma International Prognostic Index (FLIPI) with age, Ann Arbor stage, hemoglobin level (<12 g/dL), and number of nodal sites. All information was obtained from the medical records and the aforementioned data registries from prospective cohort studies. The frontline treatment was determined based on regimens reimbursed by the current National Health Insurance, although some patients received chemotherapy regardless of reimbursement. Because RCVP, RCHOP, and BR were reimbursed as frontline treatments in 2010, 2013, and 2018, respectively, the number of patients receiving RCVP was larger than that of RCHOP and BR. In particular, RCVP was more commonly used than RCHOP due to its manageable toxicity profiles, until BR reimbursement began in 2018, whereas RCHOP was preferred for patients with grade 3A or high tumor burden, at the discretion of the treating physicians. Reimbursement for rituximab maintenance therapy every two months for two years began in 2012 for stage III/IV patients who achieved a complete or partial response to frontline chemotherapies. The response was evaluated according to the current criteria for lymphoma using computed tomography (CT) and ^18^F-fluorodeoxyglucose positron emission tomography (PET)/CT scans. In clinical practice, baseline evaluations with CT and PET/CT were conducted before frontline treatment, with response evaluations performed after the third and 6th cycle of the frontline treatment. After that, CT evaluations were performed every three to four months for two years regardless of rituximab maintenance. For surveillance for relapse and survival status, patients were regularly monitored every three to six months within the first two years, and then, every six months to one year. If a patient showed evidence of disease progression on imaging studies, including a CT scan, this was counted as a progression event regardless of whether they required immediate treatment for progression. The last survival and disease status updates were collected in August 2020.

### Statistical analysis

Overall survival (OS) was defined as the time between the date of initiation of frontline chemotherapy and either the date of death for any reason or the last follow-up. PFS was defined as the date of initiation of frontline chemotherapy to the date of relapse, progression, or death for any reason. OS after progression was defined as the time between the date of the aforementioned relapse or progression and either the date of death for any reason or the last follow-up for patients experiencing progression. Survival curves were produced using the Kaplan-Meier method and compared using the log-rank test. Median follow-up was calculated using a reverse Kaplan-Meier survival curve, which was constructed by reversing “censor” and “event” 15. The associations between transformation and POD24 with clinical parameters were assessed using Fisher's exact probability test. The maximum standardized uptake value (SUVmax) of ^18^F-fluorodeoxyglucose PET/CT was compared according to the occurrence of histological and clinical transformation by the Wilcoxon signed-rank test. Two-sided P-values < 0.05 were considered statistically significant. Statistical analyses were performed using the statistical software package IBM SPSS Statistics version 24.0 (IBM Corp., Armonk, NY, USA).

## Results

### Patient characteristics

**Figure 1:**
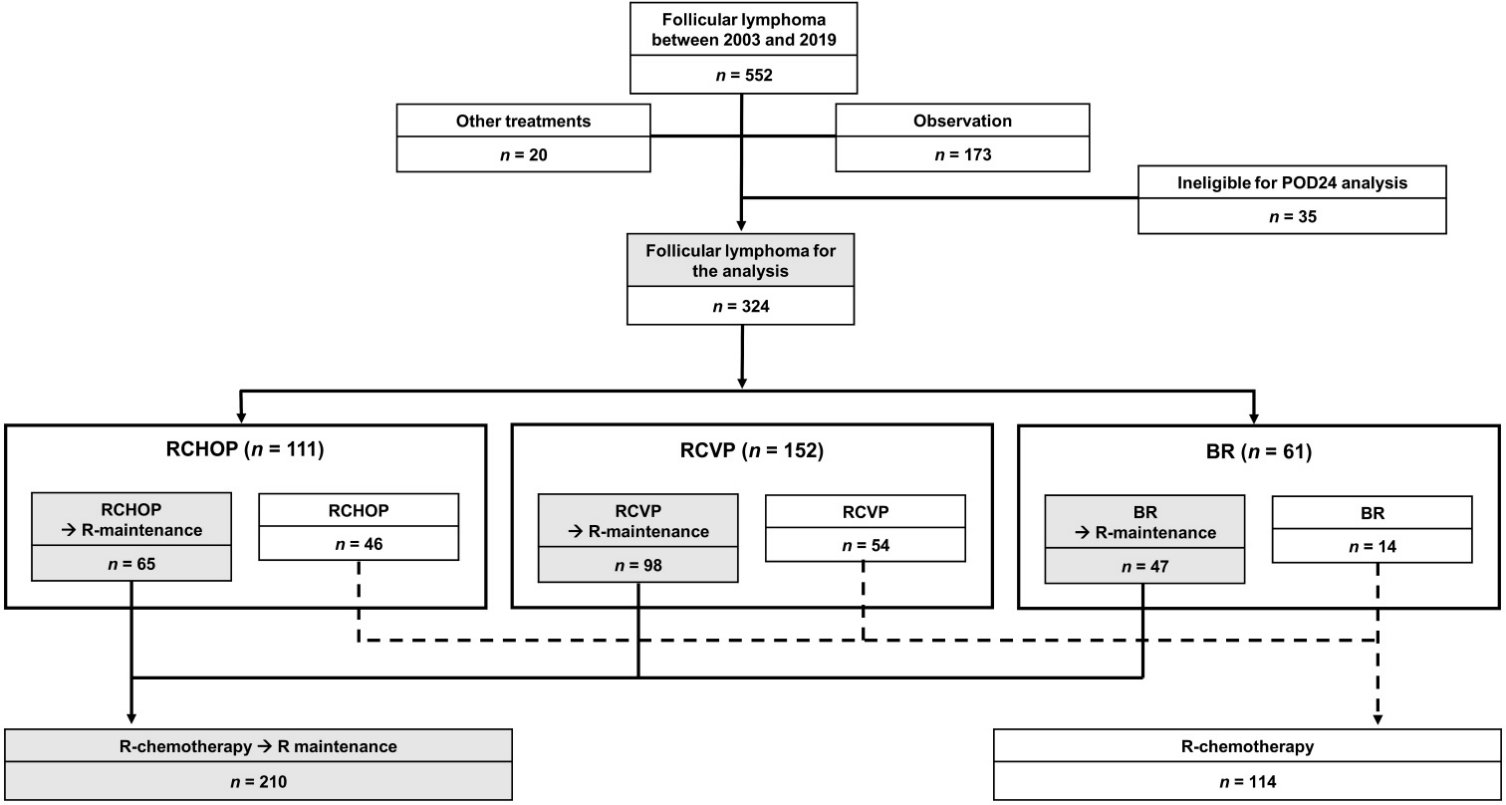
Study sample.

According to the inclusion and exclusion criteria, we excluded 20 patients who underwent other chemotherapies, 173 patients who were under observation without any chemotherapies, and 35 patients who were ineligible for POD24 analysis due to short follow-up duration. However, patients with early relapse or progression were included in this analysis even though their follow-up duration was less than 24 months. Consequently, the total sample was 324 patients. According to frontline chemotherapies, patients were grouped as RCVP (n = 152, 46.9%), RCHOP (n = 111, 34.3%), and BR (n = 61, 18.8%, Figure 1). The median patient age at diagnosis was 50 years (range: 20-84 years). Because frontline chemotherapies were begun following diagnosis in most patients, except for 12 who received delayed treatment after observation, the median age at frontline treatment was also 50 years. After completing the frontline treatment, 64.8% of patients (n = 210) received rituximab maintenance therapy (Figure 1). Patients' clinical and laboratory characteristics were compared based on the frontline treatment type (Table 1). Stage III/IV and the risk of FLIPI did not differ among groups; however, the proportion of patients with grade 3A and elevated serum β-2 microglobulin differed because RCVP was used less frequently than RCHOP or BR for patients with grade 3A (Table 1).

### Survival outcomes

**Figure 2:**
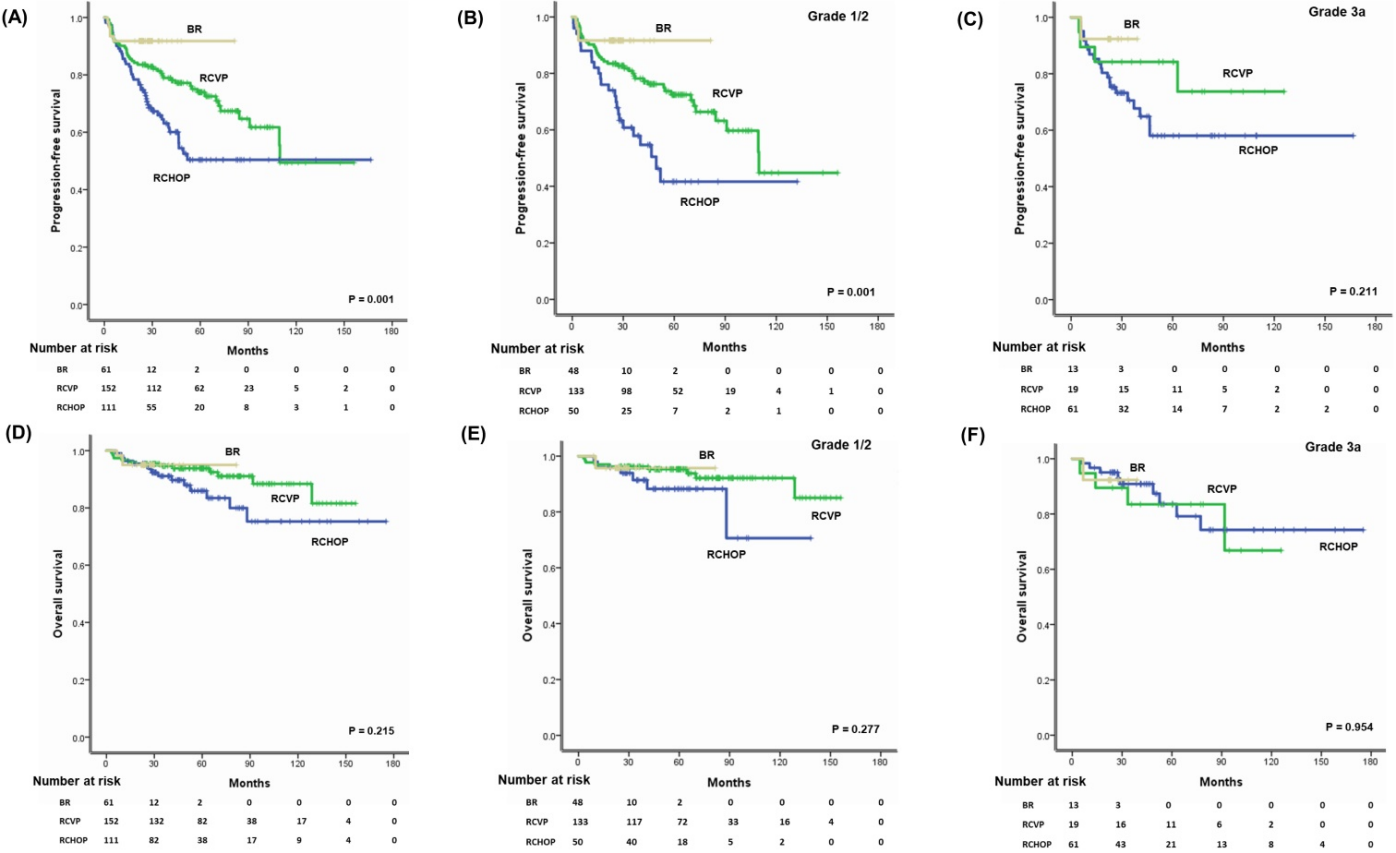
(A, B) Three-year PFS and OS of patients treated with BR, RCVP, or RCHOP. (C, D) Three-year PFS and OS in grade 1 or 2 patients. (E, F) Three-year PFS and OS of grade 3 patients.

The median follow-up for the sample was 48.3 months (95% confidence interval [CI]: 43.0-53.5 months); however, follow-up duration differed among the groups because reimbursement for each regimen differed. Thus, the median follow-up time for 61 patients receiving BR (24.5 months, 95% CI: 23.1-25.9 months) was shorter than for those receiving RCVP (64.4 months, 95% CI: 56.6-72.2 months) or RCHOP (50.1 months, 95% CI: 42.7-57.5 months). Accordingly, the number of patients who had died by the time of analysis differed among the three groups: RCHOP (15/111, 13.5%), RCVP (13/152, 8.6%), and BR (3/61, 4.9%). Although comparisons of the three treatment regimens were necessarily limited due to the study's retrospective nature and different follow-up durations, patients receiving RCHOP showed inferior PFS compared with RCVP and BR (Figure 2A). However, there was not a significant difference in OS among the three regimens (Figure 2D). Among patients with grades 1 and 2, the PFS comparison revealed worse PFS among the RCHOP group than the RCVP and BR groups; however, OS did not differ (Figure 2B, E). Comparing the three regimens in patients with grade 3A showed no significant PFS or OS differences (Figure 2C, F).

### POD24 and transformation

**Figure 3:**
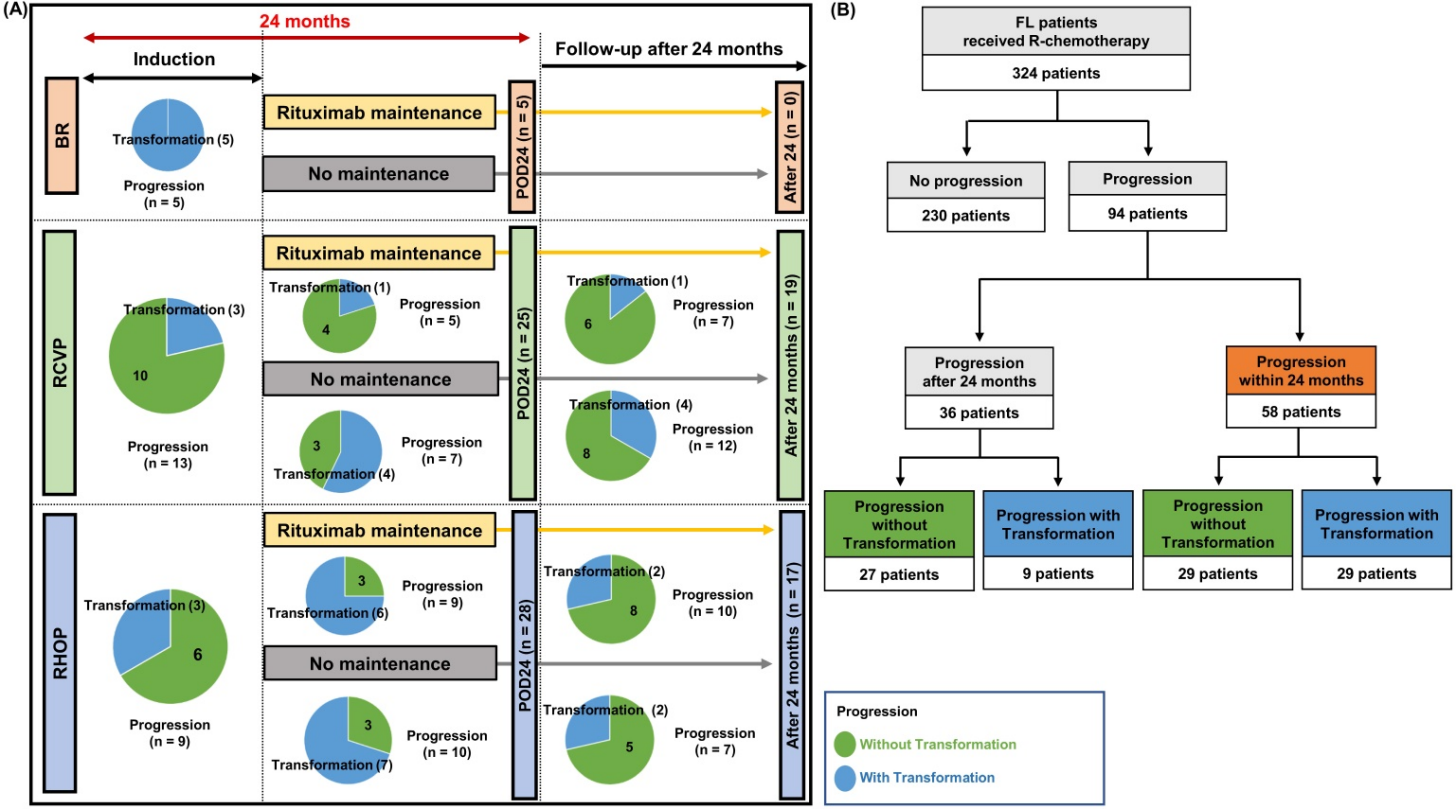
(A) Comparison of transformation and POD24 based on induction treatment type. Each circle represents the number of cases with and without transformation. Blue indicates transformation and green indicates progression without transformation. (B) Summary of transformation in patients with progression within or after 24 months.

Disease progression was found in 94 patients (94/324, 29.0%). This number was lower in the BR group (n = 5, 8.2%) than the RCVP (n = 44, 28.9%) and RCHOP (n = 45, 40.5%) groups. This difference may have been influenced by the shorter follow-up duration in the BR group than in the RCVP and RCHOP groups. POD24 occurred in 58 patients (58/324, 17.9%), and those treated with RCVP (25/152, 16.4%) or RCHOP (28/111, 25.2%) showed a higher frequency of occurrence than the BR group (5/61, 8.2%, Figure 3A). Among these 58 POD24 cases, 27 occurred during treatment induction: BR (5/61, 8.2%), RCVP (13/152, 9.1%), and RCHOP (9/111, 7.8%, Figure 3A). Among the 94 cases of progression, transformation was confirmed by biopsy in 16 patients. However, 22 cases were designated as transformation based on the aforementioned index for clinical suspicion either because they had nodal involvement in which percutaneous biopsy was not possible or because exploratory laparotomy under general anesthesia was not possible due to their rapid clinical deterioration or poor general condition. Among the 58 cases with POD24, the proportion of transformation differed across the groups: RCVP (8/25, 32%), RCHOP (16/28, 57.1%), and BR (5/5, 100%); no cases of progression or transformation occurred during rituximab maintenance in the BR group, contrary to the RCVP and RCHOP groups (Figure 3A). Accordingly, transformation accounted for 50% (29/58) of POD24, whereas only 9 cases (9/36, 25%) of transformation occurred in patients with progression after 24 months (Figure 3B).

### Survival after transformation and POD24

**Figure 4:**
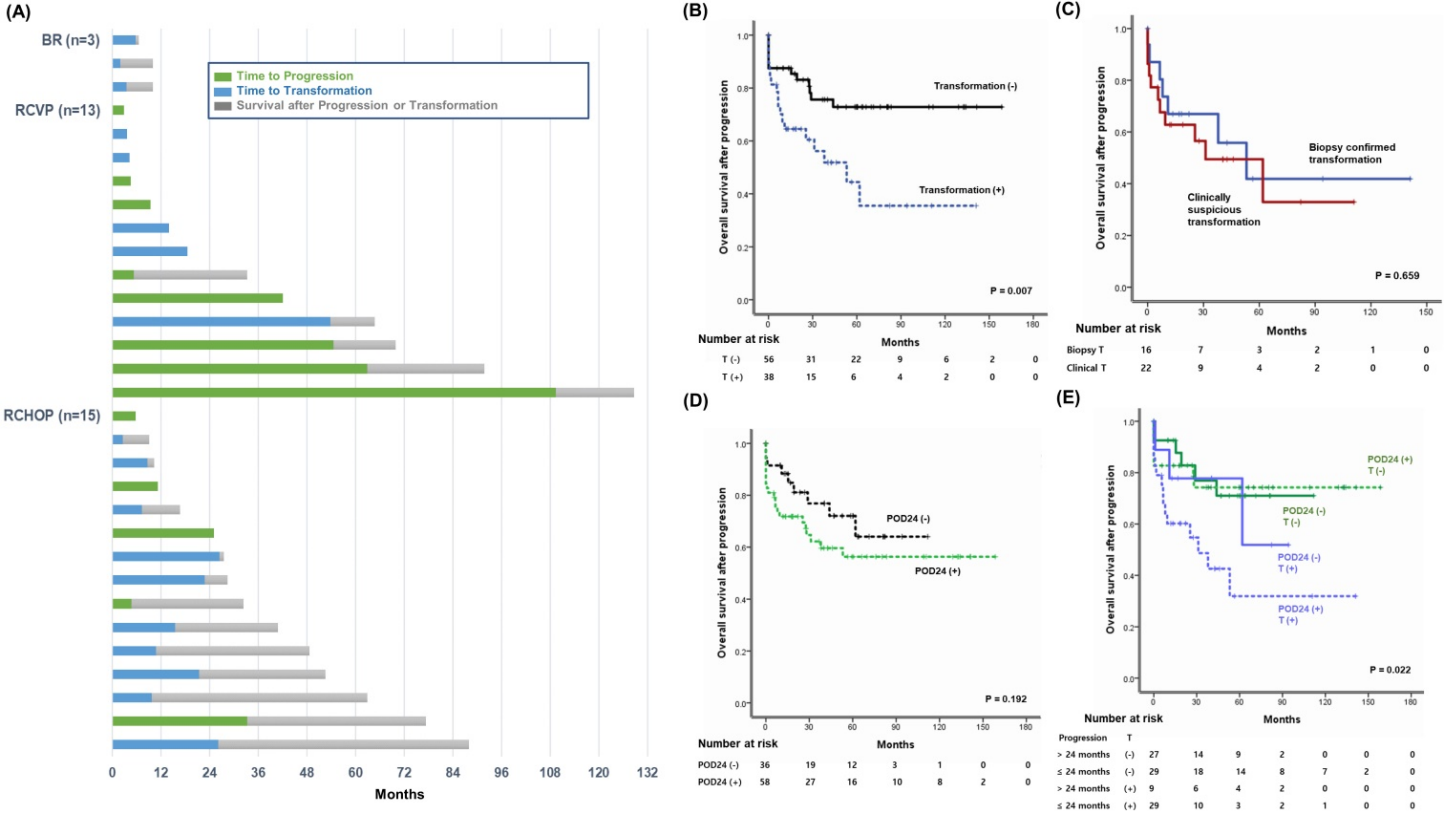
(A) Swimmer plot of 31 patients who died after progression with or without transformation. (B) Comparison of overall survival after progression according to transformation. (C) Comparison of overall survival after progression in 38 patients with transformation between biopsy-confirmed transformation (n = 16) and clinically suspicious transformation (n = 22). (D) Comparison of overall survival after progression in 94 patients with progression according to the occurrence of POD24. (E) Comparison of overall survival in 94 patients with progression: those with or without POD24 and those with transformation within or after 24 months. T: transformation.

Out of 31 patients who had died at the time of analysis, transformation was the major cause of death in the BR (3/3, 100%), RCHOP (10/15, 66.7%), and RCVP (5/13, 38.4%) groups (Figure 4A). When OS after progression was compared based on transformation occurrence among the 94 patients who experienced progression, the OS among patients with transformation was significantly worse than among patients without transformation, regardless of POD24 (Figure 4B). Among the 38 patients with transformation, the OS after transformation did not differ significantly between the 22 patients with transformation based on clinical criteria and the 16 patients with biopsy-confirmed transformation (Figure 4C). Once progression occurred, the OS after progression did not differ between patients with or without POD24 (Figure 4D). Because transformation accounted for 50% (29/58) of POD24 cases, 94 patients were classified into four groups according to the occurrence of POD24 and/or transformation. Comparing OS after progression among these four groups showed that 29 patients with transformation within 24 months had the worst survival outcome after progression, and that the remaining 65 patients' OS did not differ significantly regardless of POD24 and transformation (Figure 4E).

### Comparison of histological and clinical transformation

**Figure 5:**
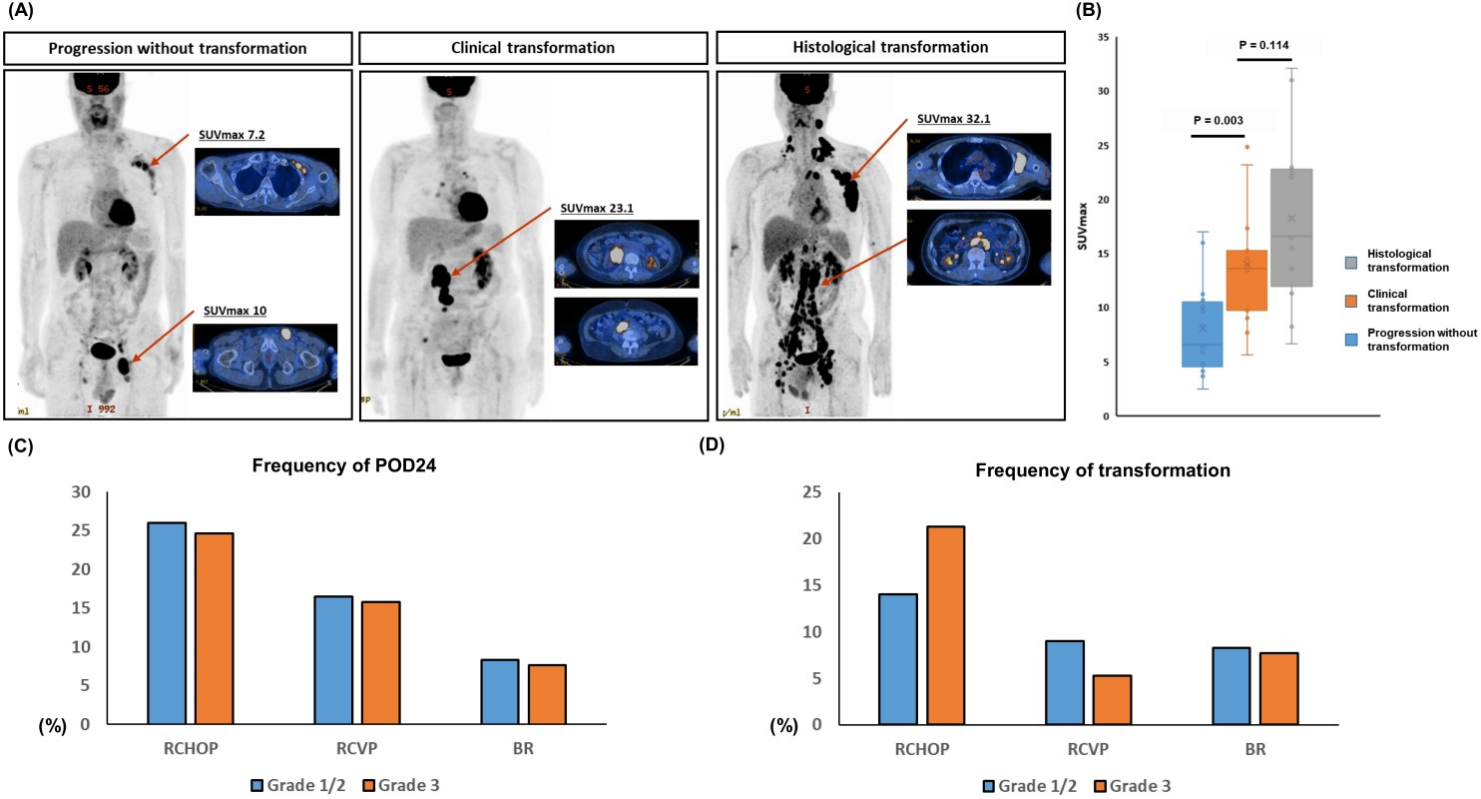
(A) Representtive images of the maximum standardized uptake value (SUVmax) of 18F-fluorodeoxyglucose PET/CT (B) The comparison of SUVmax between cases with histological and clinical transformation, and progressed cases without any evidence of transformation (C, D) The comparison of frequency of POD24 and transformation in three treatment groups according to the grade of FL.

This study used the clinical suspicion index for transformation considering the biopsy of involved sites at relapse or progression was not always possible. However, this clinical index might be insufficient compared to the histological confirmation. Thus, we analyzed the SUVmax of ^18^F-fluorodeoxyglucose PET/CT for the comparison of histological and clinical transformation because the SUVmax of representative cases with transformation showed the higher SUVmax than a progressed case without transformation (Figure 5A). When we compared the SUVmax of 12 cases with histological confirmed transformation to that of 15 cases with clinically suspicious transformation cases using the data of 27 patients whose the results of PET/CT scan were available for analysis, there was no significant difference between them (P = 0.114, Figure 5B). On the other hand, the SUVmax of progressed cases without any evidence of transformation was significantly lower than that of cases with clinical or histological transformation (P < 0.05, Figure 5B).

### Risk factor analysis for transformation and POD24

When the frequency of POD24 was compared according to the grade of FL, each treatment group did not show a significant difference although the percentage of POD24 was higher in the RCHOP group than RCVP or BR group (P > 0.05, Figure 5C). The frequency of transformation also showed the similar pattern to that of POD24, and the grade of FL did not show a significant diffece of transformation (P > 0.05, Figure 5D). The clinical and laboratory characteristics of patients with transformation showed higher frequencies of hemoglobin < 12 g/dL, elevated serum LDH, elevated β2-microglobulin, and more stage III/IV than patients without transformation (Table 2). Accordingly, a high risk of FLIPI was significantly associated with the occurrence of transformation. Risk factors for POD24 were the same as those for transformation (Table 2). On the other hand, age, B symptoms, grade 3A, involvement of five or more lymph nodes, presence of bulky mass, and bone marrow involvement were correlated with neither transformation nor POD24. Comparing the characteristics of patients with POD24 according to the occurrence of transformation failed to reveal any factors that were significantly related to transformation, although this result may have been affected by the relatively small number of cases (Table 2).

## Discussion

This study analyzed the outcomes of 324 patients who were treated with one of three frontline therapies, BR, RCHOP, or RCVP. Direct comparisons of PFS and OS among the three groups was challenging in this retrospective study because the median follow-up among 61 patients receiving BR (24.5 months) was shorter than that of RCVP (64.4 months) and RCHOP (50.1 months). Furthermore, the number of patients with grade 3A was higher in the RCHOP group because anthracycline-containing chemotherapy was preferred for patients with grade 3A FL in the past 16, 17. Nevertheless, our results supporting the superior PFS of BR to that of RCVP and RCHOP were consistent with a recent result from the BRIGHT 5-year follow-up study, which reported a 65.5% higher 5-year PFS rate in the BR group than in the RCHOP/RCVP group 18. Indeed, a recent European multicenter retrospective study demonstrated the superior outcome of BR to RCHOP in terms of PFS and toxicity profiles for patients with grade 3A FL 19.

At the time of analysis, we found 94 cases with progression, POD24 occurred in 58 (58/324, 17.9%), and transformation was found in 38 (38/324, 11.7%). In previous studies, the incidence of POD24 was 19% in FL patients who received RCHOP, and 13% in patients treated with BR 10, 20. Our overall incidence of transformation was comparable to that of the previous JCOG0203 trial comparing RCHOP21 and RCHOP14 for patients with grades 1-3 FL, reporting a cumulative incidence of 8.5% at eight years 21. In our study, the majority of transformation cases (29/38, 76.3%) were patients with POD24, among whom transformation accounted for 50% (29/58) of POD24 and 9 cases with transformation were found in 36 patients experiencing late progression after 24 months (Figure 3A, B). Thus, when the frequency of POD24 was compared based on the frontline treatment type, the occurrence of POD24 was significantly less frequent in the BR group (5/61, 8.2%) than the RCVP (25/152, 16.4%) and RCHOP (28/111, 25.2%) groups. However, the proportion of transformation was significantly lower in the RCVP (8/152, 5.2%) group than in the BR (5/61, 8.2%) and RCHOP (16/111, 14.4%) groups because all cases of POD24 harbored transformation in the BR group (Figure 3A). These results implied that BR treatment could reduce the incidence of POD24, whereas early progression after BR treatment may have a high probability of transformation. Indeed, a recent retrospective analysis of a population-based cohort of 296 patients with FL treated with frontline BR followed by rituximab maintenance reported that early progression after BR treatment was related to transformation and that the presence of early transformation could be the main cause of POD24 20.

In our survival analysis of 94 patients with progression, transformation negatively impacted OS after progression regardless of the frontline treatment type (Figure 4B). Furthermore, the OS among patients with late progression after 24 months did not differ significantly from POD24 patients without transformation or patients with late transformation after 24 months. Thus, patients with early transformation within 24 months after the initiation of frontline treatment showed the worst OS (Figure 4E). Thus, whether patients with POD24 have related transformation may be especially important for treatment decisions. Herein, 16 cases with transformation were confirmed by biopsy and 22 cases of transformation were determined by clinical criteria. Transformation should be confirmed according to histological findings from a biopsy because transformation cases could show the transformation to DLBCL, or lymphomas resembling BL or the intermediate between DLBCL and BL 13. However, a biopsy is not always possible and, indeed, the PRIMA study found that only 42% of patients were biopsied at the time of the first relapse 22. Thus, transformation designation based on a high index of suspicion could help manage patients with FL with progressive disease, especially in early progression cases. Indeed, previous population-based analyses of incidence and outcome of transformation in patients with FL reported that clinically diagnosed transformation has an equivalent impact on outcome compared with biopsy-proven transformation 14. Likewise, our OS comparison showed no significant difference between biopsy-confirmed and clinically determined transformation (Figure 4C), and the SUVmax of PET/CT scan was not significantly different between clinical and hostolgocial transformation (Figure 5A, B).

Our study showed that hemoglobin < 12 g/dL, increased LDH, elevated β2-microglobulin, stage III/IV, and high FLIPI score were significantly associated with transformation occurrence, whereas grade 3A and B symptoms were not related to transformation (Table 2). These risk factors were also associated with POD24 because transformation was strongly associated with POD24. Thus, if patients showed suspicious clinical signs of transformation, such as rapidly increasing tumor mass or rising serum LDH, a thorough evaluation for transformation and appropriate subsequent management should be carried out, especially in patients with the aforementioned high risk of transformation (i.e., hemoglobin < 12 g/dL, increased LDH, elevated β2-microglobulin, and high FLIPI score at diagnosis).

In conclusion, transformation occurrence negatively impacts OS among patients with FL more than POD24, regardless of frontline treatment type. With caution, our results suggest that, compared with RCVP and RCHOP, BR may improve treatment outcomes among patients with grades 1-3A FL by reducing POD24 whereas BR may not be efficacious for reducing transformation occurrence. However, these findings should be confirmed by future studies because the direct comparison of three regimens was not possible in this study due to the nature of retrospective study. Furthermore, there was the major limitation of suggesting the impact of transformation on the outcome of FL patients because our analysis included cases with clinically suspicious transformation. Considering the limited number of our transformation cases that were biopsy-confirmed, future studies should focus on pathologically confirmed transformation cases, using a prospective design, with a more extensive study sample.

## Figures and Tables

**Table 1:** Patient characteristics at diagnosis (n = 324)

Parameters	Total	RCHOP	RCVP	BR	*P*-value
	324 (100)	111 (34.3)	152 (46.9)	61 (18.8)	
**Age (n, %)**					
≤ 60	254 (78.4)	88 (79.3)	121 (79.6)	45 (73.8)	0.62
> 60	70 (21.6)	23 (20.7)	31 (20.4)	16 (26.2)	
**Sex (n, %)**					
Male	161 (49.7)	62 (55.9)	72 (47.4)	27 (44.3)	0.26
Female	163 (50.3)	49 (44.1)	80 (52.6)	34 (55.7)	
**ECOG PS (n, %)**					
0/1	313 (96.6)	108 (97.3)	146 (96.1)	59 (96.7)	0.86
≥ 2	11 (3.4)	3 (2.7)	6 (3.9)	2 (3.3)	
B-symptom (n, %)	26 (9.6)	11 (9.9)	12 (7.9)	3 (4.9)	0.65
Anemia < 12 g/dL (n, %)	52 (16.0)	22 (19.8)	19 (12.5)	11 (18.0)	0.25
Elevated LDH (n, %)	60 (18.5)	25 (22.5)	23 (15.1)	12 (19.7)	0.30
Elevated β2-microglobulin (n, %)	79 (24.4)	33 (29.7)	29 (19.1)	17 (27.9)	0.007
Bulky mass (n, %)	26 (8.0)	11 (9.9)	11 (7.2)	4 (6.6)	0.66
Bone marrow involvement (n, %)	156 (48.1)	51 (45.9)	70 (46.1)	35 (57.4)	0.07
Nodal site ≥ 5 (n, %)	143 (44.1)	44 (39.6)	64 (42.1)	35 (57.4)	0.06
**Ann Arbor stage (n, %)**					
I/II	71 (21.9)	22 (19.8)	39 (25.7)	10 (16.4)	0.27
III/IV	253 (78.1)	89 (80.2)	113 (74.3)	51 (83.6)	
**FLIPI (n, %)**					
Low risk (0-1)	129 (39.8)	41 (36.9)	69 (45.4)	19 (31.1)	0.22
Intermediate risk (2)	114 (35.2)	37 (33.3)	52 (34.2)	25 (41.0)	
High risk (3-5)	81 (25.0)	33 (29.7)	31 (20.4)	17 (27.9)	
**Grade (n, %)**					
1	163 (50.3)	34 (30.6)	100 (65.8)	29 (47.5)	<0.001
2	68 (21.0)	16 (14.4)	33 (21.7)	19 (31.1)	
3a	93 (28.7)	61 (55.0)	19 (12.5)	13 (21.3)	

Abbreviations: ECOG PS, Eastern Cooperative Oncology Group performance status; LDH, lactate dehydrogenase FLIPI, Follicular Lymphoma International Prognostic Index.

**Table 2:** Risk factor analysis for transformation and POD24

	Transformation			POD24			Transformation in POD24		
	Not occurred	Occurred	*P*	Not occurred	Occurred	*P*	POD24	T + POD24	*P*
Parameters, N (%)	286 (88.3)	38 (11.7)		266 (82.1)	58 (17.9)		29 (50)	29 (50)	
Age ≥ 60 years	58 (20.3)	12 (31.6)	0.140	57 (21.4)	13 (22.4)	0.861	5 (17.2)	8 (27.6)	0.530
B-symptoms	23 (8.0)	3 (7.9)	0.182	19 (7.1)	7 (12.1)	0.415	4 (13.8)	3 (10.3)	0.375
Hemoglobin < 12 g/dL	38 (13.3)	14 (36.8)	0.001	37 (13.9)	15 (25.9)	0.030	4 (13.8)	11 (37.9)	0.070
Elevated LDH	47 (16.4)	13 (34.2)	0.013	40 (15.0)	20 (34.5)	0.001	9 (31)	11 (37.9)	0.783
Elevated β2m	65 (22.7)	14 (36.8)	0.004	58 (21.8)	21 (36.2)	0.006	10 (34.5)	11 (37.9)	0.477
Nodal site ≥ 5	127 (44.4)	16 (42.1)	0.863	114 (42.9)	29 (50.0)	0.381	16 (55.2)	13 (44.8)	0.600
Bulky mass	23 (8.0)	3 (7.9)	> 0.999	21 (7.9)	5 (8.6)	0.793	2 (6.9)	3 (10.3)	>0.999
BM involvement	140 (49.0)	16 (42.1)	0.301	126 (47.4)	30 (51.7)	0.615	17 (58.6)	13 (44.8)	0.168
Grade 3A	78 (27.3)	15 (39.5)	0.129	74 (27.8)	19 (32.8)	0.522	6 (20.7)	13 (44.8)	0.092
FLIPI ≥ 3	65 (22.7)	16 (42.1)	0.020	58 (21.8)	23 (39.7)	0.005	11 (37.9)	12 (41.4)	0.446
Stage III/IV	218 (76.2)	35 (92.1)	0.035	201 (75.6)	52 (89.7)	0.022	26 (89.7)	26 (89.7)	>0.999

Abbreviations: POD24, progression of disease within 24 months; LDH, lactate dehydrogenase; β2m, β2-microglobulin; BM, bone marrow; FLIPI, Follicular Lymphoma International Prognostic Index; T, transformation.
